# ﻿The genus *Blepharicera* Macquart, 1843 newly recorded from Sichuan, China with descriptions of three new species (Diptera, Blephariceridae)

**DOI:** 10.3897/zookeys.1085.75885

**Published:** 2022-02-04

**Authors:** Xiao Zhang, Zehui Kang

**Affiliations:** 1 Key Lab of Integrated Crop Pest Management of Shandong Province, College of Plant Health and Medicine, Qingdao Agricultural University, Qingdao 266109, China Qingdao Agricultural University Qingdao China

**Keywords:** Blepharicerinae, chinese fauna, net-winged midge, taxonomy

## Abstract

The genus *Blepharicera* Macquart, 1843 is recorded from Sichuan, China for the first time with the following three new species: *B.gengdica***sp. nov.**, *B.balangshana***sp. nov.** and *B.kongsica***sp. nov.**, increasing the number of Chinese *Blepharicera* species to eleven. The new species are distinguished from congeners mainly by their male genitalia. Descriptions and illustrations for the new species and an updated key to Chinese *Blepharicera* species are presented.

## ﻿Introduction

Family Blephariceridae, also called the net-winged midge, is a kind of slender delicate fly in lower Diptera. Compound eyes of blepharicerids are transversely divided into dorsal divisions and ventral divisions. Mandibles are absent in males and present in most females. Wings of blepharicerids have a net-like pattern of folds in the wing membrane. Larvae and pupae are often found on rocks in swiftly moving streams or waterfalls (Hogue 1981). Adults are usually found close to the natal stream resting on vegetation or logs (Courtney 2000a).

Blephariceridae is considered a small family with approximately 320 described species in 28 genera (Jacobson et al. 2011). Seven genera of Blephariceridae are known to occur in China (Kitakami 1931, 1950; Mannheims 1938; Kang and Yang 2012, 2014, 2015). *Agathon* Röder, 1890, *Bibiocephala* Osten Sacken, 1874 and *Neohapalothrix* Kitakami, 1938 are found in northeast China, *Apistomyia* Bigot, 1862 in Taiwan, *Horaia* Tonnoir, 1930 in southwest China, and *Philorus* Kellogg, 1903 in three provinces of China (Hebei, Sichuan and Taiwan). *Blepharicera* Macquart, 1843 is the most widely distributed genus of blepharicerids recorded from China, in seven provinces in both south and north China. Eight species of *Blepharicera* are known to occur in China (Kang and Yang 2014): *B.asiatica* (Brodsky, 1930) distributed in Yunnan and Guangxi Provinces, *B.dimorphops* Alexander, 1953 in Fujian, *B.hainana* Kang & Yang, 2014 and *B.macropyga* Zwick, 1990 from Hainan, *B.hebeiensis* Kang & Yang, 2014 in Heibei and Shanxi, *B.taiwanica* Kitakami, 1937 and *B.uenoi* Kitakami, 1937 in Taiwan, and *B.yamasakii* Kitakami, 1950 in Heilongjiang.

*Blepharicera* can be easily distinguished from other genera of Blephariceridae by the following features: head normally dichoptic in male and subholoptic in female; antennae with 13 flagellomeres; middle coxa of female with setose median outgrowth; base of hind basitarsus with obvious black setae; claws nonsetate dorsally; vein R with 3 branches, veins R_4_ and R_5_ separate for entire length; absence of cross vein bm-cu, presence of M_2_ (Zwick 1990; Courtney 2000b; Jacobson et al. 2011).

Sichuan province is situated in southwestern China; it includes Sichuan Basin, and parts of the Qinghai-Tibetan Plateau and the Hengduan Mountain region, which has been designated as one of the world’s biodiversity hotspots (Zhang and Ma 2008; Zhang et al. 2009). As a region of high biodiversity, however, the blepharicerid species in this area has been poorly described. Only one species belonging to *Philorus* was described by Kang and Yang (2012).

**Figure 1.**
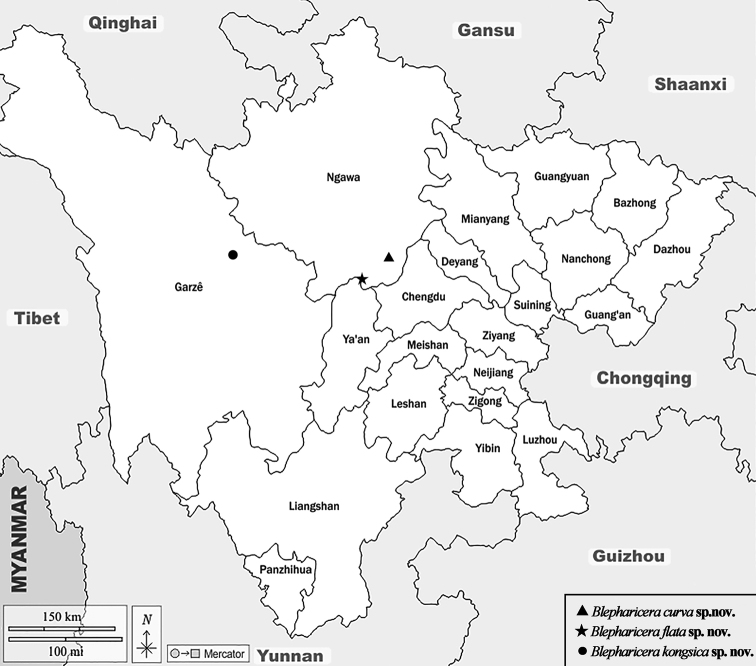
Distribution map of *Blepharicera* from Sichuan.

Several insect diversity investigations in Sichuan Province were initiated by the authors and other entomologists from 2013 to 2016, and the genus *Blepharicera* was found in Sichuan province for the first time (Fig. 1). In this paper, descriptions and illustrations for three new species from Sichuan, *B.gengdica* sp. nov., *B.balangshana* sp. nov. and *B.kongsica* sp. nov. are provided, and a key to Chinese *Blepharicera* species modified from Kang and Yang (2014) is also presented.

## ﻿Material and methods

Adults were collected by insect net and light trap. Type specimens of the new species in this study were deposited in the Entomological Museum of China Agricultural University, Beijing, China (CAU) and the Entomological Museum of Qingdao Agricultural University, Shandong, China (QAU). Studies were based on whole-animal preparations and dissections. Photographs were captured by a Canon EOS 90D digital camera through a macro lens. Genitalia were prepared by immersing the apical portion of the abdomen in warm lactic acid for 0.5–1 hours. Specimens were examined and illustrations prepared by using a ZEISS Stemi 2000-C stereomicroscope. After examination, the removed abdomen was transferred to fresh glycerine and stored in a microvial pinned to the respective specimen. Structural terminology is based primarily on Courtney (2000b).

## ﻿Taxonomy

### ﻿Key to adult males of Chinese species of *Blepharicera*

Adult unknown in *B.uenoi* Kitakami

**Table:** 

1	Dorsal division of compound eye large, at least 1/2 of ventral division (Fig. 5a, b)	**2**
–	Dorsal division of compound eye small, at most 1/10 of ventral division (Figs 3a, b, 8a, b)	**5**
2	Gonostylus bifurcated	**3**
–	Gonostylus not bifurcated	**4**
3	Ultimate flagellomere shorter than penultimate flagellomere; Rs 1.5 times as long as r-m; ventral branch of gonostylus glabrous (Kitakami 1937: figs 7, 8)	***B.taiwanica* (Taiwan)**
–	Ultimate flagellomere longer than penultimate flagellomere; Rs as long as or slightly longer than r-m; ventral branch of gonostylus with two tufts of short dense setae (Kang &Yang 2014: figs 23, 24)	***B.macropyga* (Hainan)**
4	Dorsal division of compound eye as large as ventral division (Fig. 5a, b); epandrium trapeziform, posterior margin concave; cercus triangular; gonostylus without a semicircular inside lobe near base (Fig. 5a, b)	***B.balangshana* sp. nov. (Sichuan)**
–	Dorsal division of compound eye 1/2 as large as ventral division; epandrium semicircular, posterior margin rounded; cercus semi-elliptical; gonostylus with a semicircular inside lobe near base (Kang and Yang 2014: figs 14, 15)	***B.hainana* (Hainan)**
5	Cercus triangular, posterior margin tapered medially (Figs 3c, 5c, 8c)	**6**
–	Cercus semicircular or semi-elliptical, posterior margin round medially	**8**
6	Outer gonocoxal lobe straight	***B.asiatica* (Yunnan, Guangxi; Afghanistan; India; Pakistan; Russia; Sri Lanka)**
–	Outer gonocoxal lobe S-shaped	**7**
7	Ultimate flagellomere shorter than penultimate flagellomere (Fig. 8a); dorsal branch of gonostylus broader than ventral branch (Fig. 8c); inner gonocoxal lobe fusiform (Fig. 8c, d); dorsal carina inapparent (Fig. 8f)	***B.kongsica* sp. nov. (Sichuan)**
–	Ultimate flagellomere longer than penultimate flagellomere (Fig. 3a); dorsal branch of gonostylus as broad as ventral branch (Fig. 3c); inner gonocoxal lobe digitiform (Fig. 3c, d); dorsal carina apparent (Fig. 3f)	***B.gengdica* sp. nov. (Sichuan)**
8	Mid coxa with a conical projection, conical projection about half as long as trochanter and densely with stiff black bristles towards tip (Kitakami 1950: fig. 49)	***B.yamasakii* (Heilongjiang)**
–	Mid coxa without projection like above	**9**
9	Posterior margin of epandrium not distinctly concaved medially; cercus semicircilar; gonostylus bifurcated and strongly notched apically (Kang and Yang 2014: figs 8, 9, 11)	***B.dimorphops* (Fujian)**
–	Posterior margin of epandrium concave medially, V-shaped; cercus semi-elliptical; gonostylus not bifurcated and slightly notched apically (Kang and Yang 2014: figs 18, 19, 20)	***B.hebeiensis* (Hebei, Shanxi)**

#### ﻿ Blepharicera gengdica sp. nov.

Taxon classificationAnimaliaDipteraBlephariceridae

9EA94DB6-AF1F-52BE-944E-F7F8348C6EC8

http://zoobank.org/C46F8572-AFF6-45C0-A8D8-5783D5B8015B

Figs 2 , 3

##### Diagnosis.

Compound eye with dorsal division 1/20 as large as ventral division in male. Rs 1.5 times as long as r-m. Cercus triangular. Dorsal branch of gonostylus short; ventral branch longer and broader than dorsal branch, round apically. Outer gonocoxal lobe transparent, S-shaped; inner gonocoxal lobe digitiform. Dorsal carina apparent, tip slightly blunt.

##### Description.

**Male.** Body length 4.50 mm, wing length 5.75 mm, wing width 2.00 mm.

**Figure 2.**
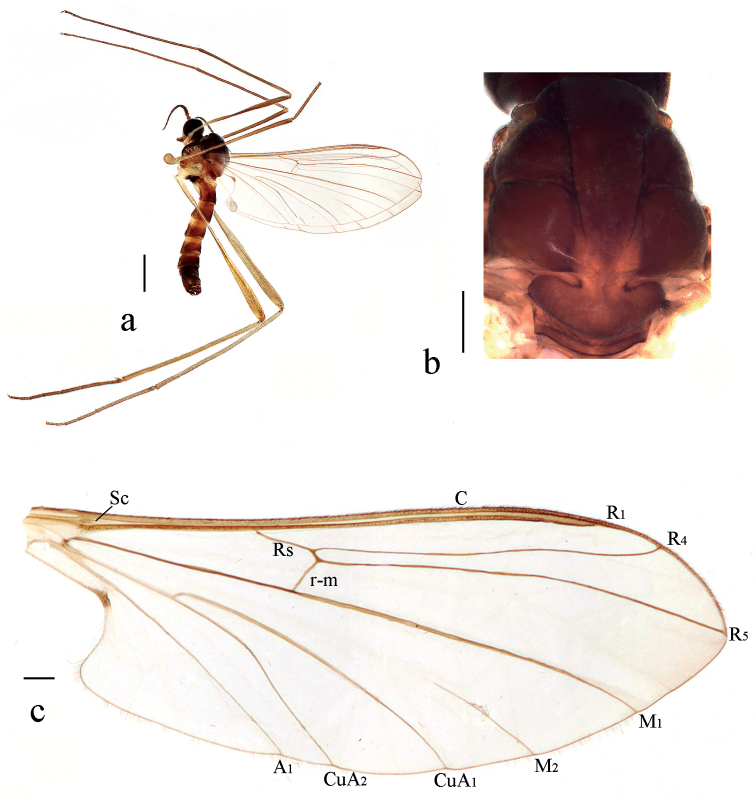
*Blephariceragengdica* sp. nov. **a** habitus of male, lateral view **b** thorax, dorsal view **c** wing. Scale bars: 1.0 mm (**a**); 0.25 mm (**b, c**).

**Figure 3.**
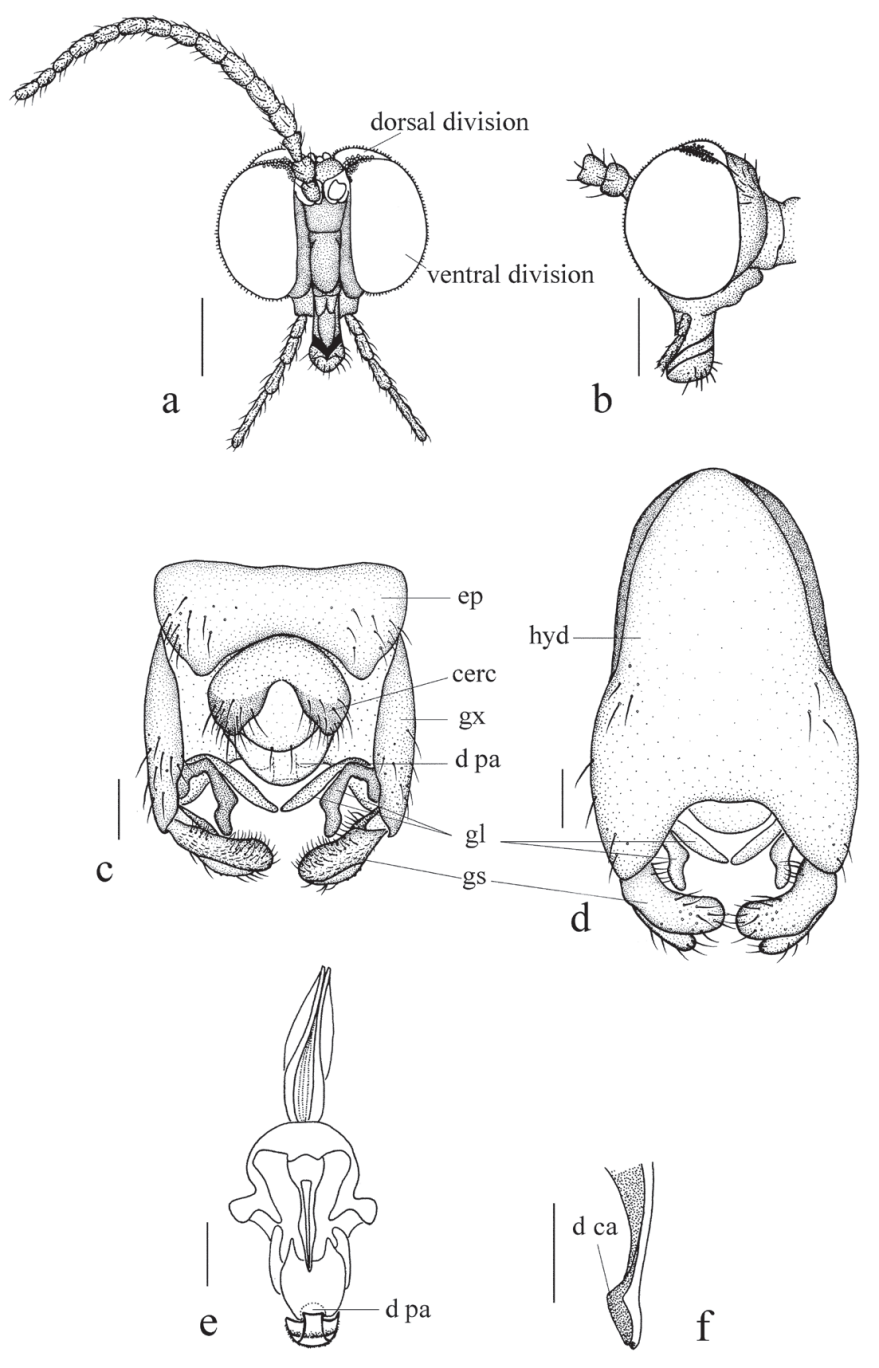
*Blephariceragengdica* sp. nov. **a** male head, frontal view **b** male head, lateral view **c** male genitalia, dorsal view **d** male genitalia, ventral view **e** aedegal complex, dorsal view **f** tip of dorsal paramere, lateral view. Scale bars: 0.25 mm (**a, b**); 0.10 mm (**c–f**). Abbreviations: cerc = cercus; d ca = dorsal carina; d pa = dorsal paramere; ep = epandrium; gl = gonocoxal lobe; gs = gonostylus; gx = gonocoxite; hyd = hypandrium.

***Head*** (Figs 2a, 3a, b) pruinose, uniformly brownish black with black hairs. Compound eyes dichoptic, interocular ridge absent; each compound eye divided, callis oculi absent; dorsal division contiguous with ventral division, 1/20 as large as ventral division; dorsal division with 6–7 rows of ommatidia, ommatidia red-orange, larger in diameter, with omatrichia; ventral division black with omatrichia. Ocelli black. Scape and pedicel oval, brown with dark brown hairs; first flagellomere conical, basal 1/2 light brown, apical 1/2 brown, with brownish black hairs; other flagellomeres cylindrical, brown with brownish black hairs; ultimate flagellomere 1.3 times length of penultimate flagellomere. Clypeus oval, brownish black, twice as long as the width; labrum brown; labellum brown with brownish black hairs; proboscis about 0.67 times length of head width. Palpus with five segments, 1^st^ segment almost invisible; 2^nd^ and 3^rd^ segments cylindrical, brownish yellow with brown hairs; 4^th^ segment cylindrical, slightly swollen apically, brownish yellow with brown hairs; 5^th^ segment slender, brownish yellow with brown hairs; relative length of distal four segments as 1.0: 1.2: 1.5: 2.9.

***Thorax*** (Fig. 2b) pruinose. Pronotum and propleuron brown without hairs. Mesonotum dark brown with middle area of posterior margin light brown; scutellum dark brown with middle area light brown, with numerous hairs grouped at posterolateral corner; episternum dark brown; anepimeron light brown, katepimeron dark brown. Relative length of femur, tibiae and 1^st^ to 5^th^ tarsomeres in fore leg as 15: 15: 10.5: 4.3: 2.8: 1.3: 1, in mid leg as 15.5: 14.5: 9.0: 4.0: 2.5: 1: 1, in hind leg as 19.6: 17.6: 7.4: 2.4: 1.6: 1: 1. Fore coxa dark brown with brown hairs; mid and hind coxae pale with brownish black hairs; trochanters pale, anterior margin with black spot apically, with brownish black hairs; fore and mid femora light yellow basally and gradually darkened to dark brown apically, with brownish black hairs; hind femur light yellow basally and gradually darkened to brownish yellow apically, with brownish black hairs; fore and mid tibiae dark brown with brownish black hairs; hind tibia brownish yellow with brown hairs; tarsomeres dark brown with brownish black hairs; claw dark brown. Tibial spurs 0–0–0. Wing (Fig. 2c) slightly brown apically, apical 1/3 of sc brown; veins brown. Sc rudimentary, not ending at base of Rs; Rs straight, 1.5 times as long as r-m; R_4_ wavy, the length from end of R_1_ to end of R_4_ shorter than length from end of R_4_ to end of R_5_; r-m straight, including angle between r-m and Rs less than 90 degrees; the length from end of M_1_ to end of M_2_ longer than the length from end of M_2_ to end of CuA_1_. Base of halter pale, apex of halter grey with brownish black hairs.

***Abdomen*.** First tergum dark brown with middle area pale, 2^nd^ tergum dark brown, 3^rd^ to 5^th^ terga dark brown with basal 1/3 brownish yellow, 6^th^ to 8^th^ terga dark brown; 1^st^ to 7^th^ sterna brownish yellow with brownish black stripes laterally; abdomen with brownish black hairs. Male genitalia (Fig. 3c–f) dark brown. Epandrium trapeziform, posterior margin concaved medially, with several brown hairs. Cercus triangular, inner margin bulge, with several brown hairs; anal cone round with two long hairs apically. Gonostylus bifurcated, dorsal branch short, slightly swollen apically, with hairs; ventral branch longer and broader than dorsal branch, round apically, with long hairs. Gonocoxal lobe bifurcated, outer gonocoxal lobe transparent, S-shaped, round apically; inner gonocoxal lobe digitiform, transparent. Hypandrium nearly triangular, twice as long as the width, round and slightly narrow basally, middle of each lateral margin slightly concave, posterior margin concave, with several brown hairs laterally. Dorsal paramere with posterior margin round; dorsal carina apparent, tip slightly blunt.

**Female**. Unknown.

##### Type material.

***Holotype***: male (CAU), China: Sichuan Province, Wenchuan County, Gengda, Fuyuan inn (Light trap), 2016.V.24, Zehui Kang.

##### Distribution.

Currently known only from China (Sichuan).

##### Etymology.

The specific name refers to the type locality Gengda.

##### Remarks.

This new species is very similar to *B.parva* Zwick & Arefina, 2005 from the Russian Far East but can be separated by the cercus being tapered posteriorly and the outer gonocoxal lobe being S-shaped. In *B.parva*, the cercus is round, and the outer gonocoxal lobe is digitiform (Zwick and Arefina 2005). This new species is also similar to *B.yamasakii* from China, but it can be separated from the latter by the mid coxa without hairy projection in male, and the triangular cercus. In *B.yamasakii*, the mid coxa has a conical projection in the male which is about half as long as trochanter and has densely stiff black bristles towards tip, and the cercus is semicircular (Kitakami 1950).

#### ﻿ Blepharicera balangshana sp. nov.

Taxon classificationAnimaliaDipteraBlephariceridae

27B98F14-E29C-5062-8525-2E5D738D387A

http://zoobank.org/5A4EDC48-D0B5-48B9-819E-A5796ADF6C02

Figs 4 , 5 , 6

##### Diagnosis.

Compound eye with dorsal division as large as ventral division in the male. Scutellum pale brown with anterior margin yellow. Rs as long as r-m. Cercus triangular. Gonostylus slightly swollen and notched apically. Dorsal carina apparent, tip nearly perpendicular. Genital fork X-shaped in female.

##### Description.

**Male.** Body length 4.50–5.00 mm, wing length 6.00–6.50 mm, wing width 2.00–2.50 mm.

**Figure 4.**
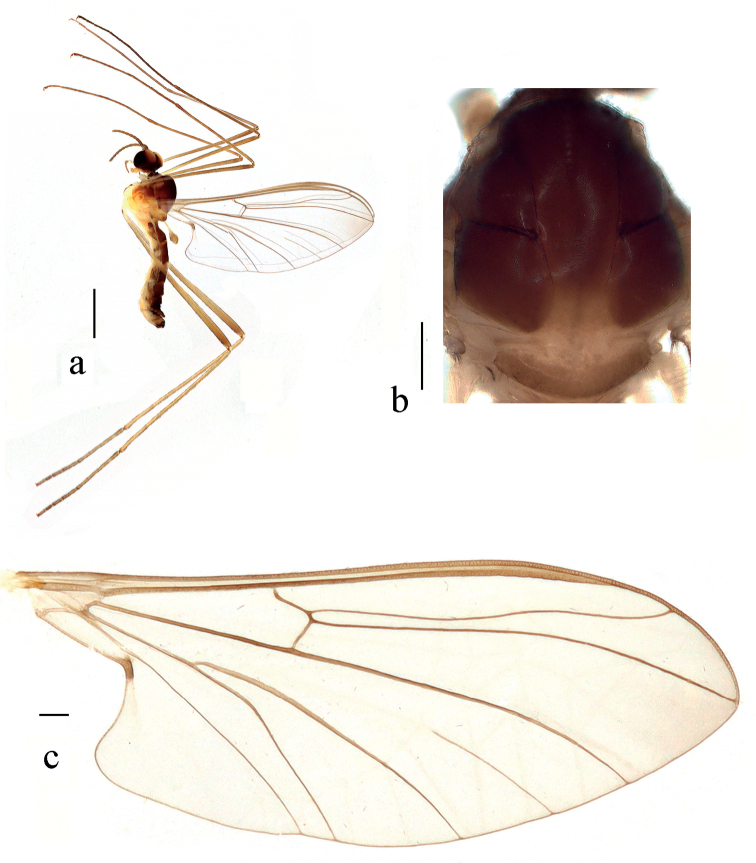
*Blepharicerabalangshana* sp. nov. **a** habitus of male, lateral view **b** thorax, dorsal view **c** wing. Scale bars: 1.0 mm (**a**); 0.25 mm (**b, c**).

**Figure 5.**
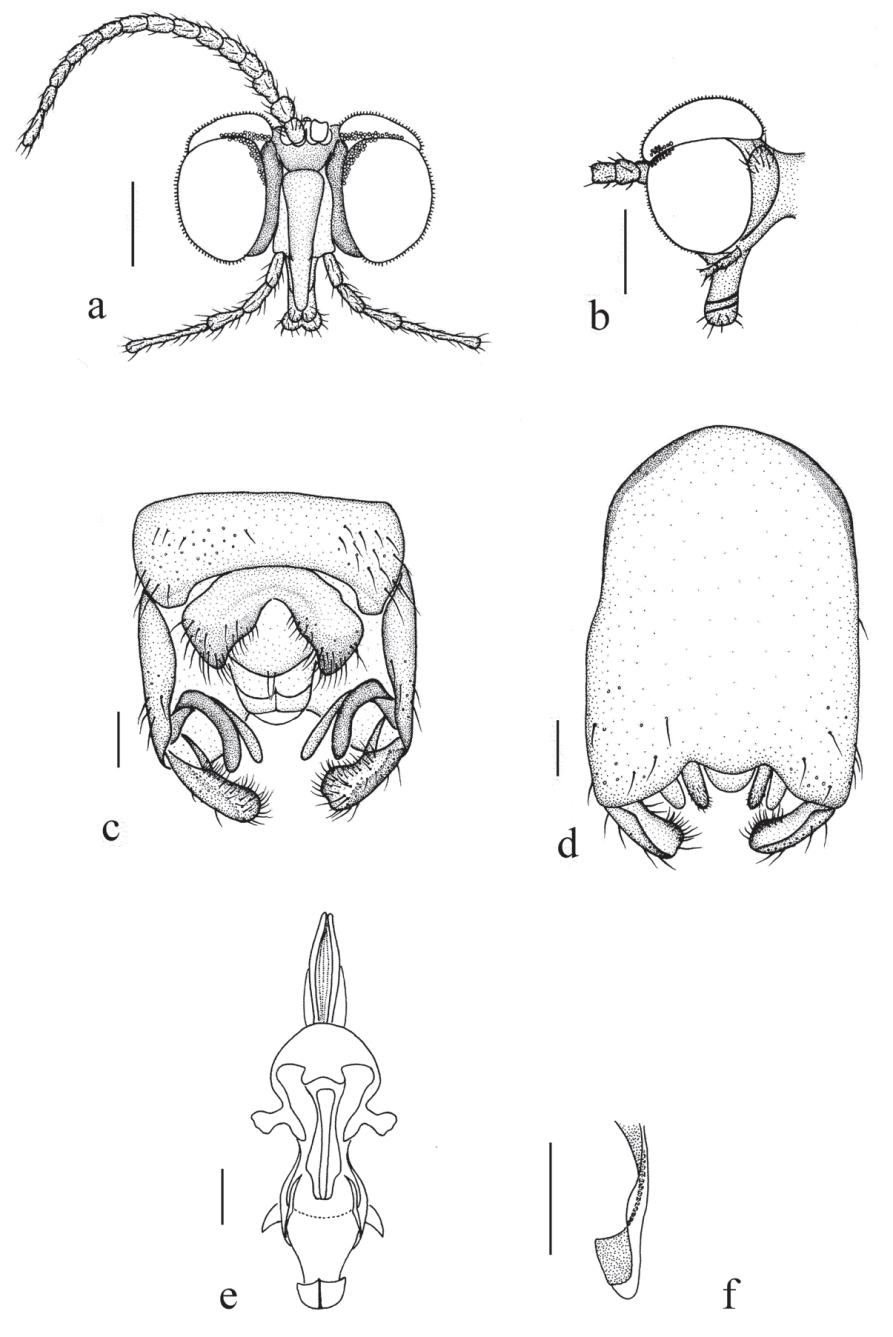
*Blepharicerabalangshana* sp. nov. **a** male head, frontal view **b** male head, lateral view **c** male genitalia, dorsal view **d** male genitalia, ventral view **e** aedegal complex, dorsal view **f** tip of dorsal paramere, lateral view. Scale bars: 0.25 mm (**a, b**); 0.10 mm (**c–f**).

***Head*** (Figs 4a, 5a, b) pruinose, uniformly brown with dark brown hairs. Compound eyes dichoptic, interocular ridge absent; each compound eye divided, callis oculi absent; dorsal division contiguous with ventral division, as large as ventral division; dorsal division with 20 rows of ommatidia, ommatidia red-orange, larger in diameter, with omatrichia; ventral division black with omatrichia. Ocelli brownish yellow. Scape and pedicel oval, brown with brownish black hairs; first flagellomere constricted at base, flared at apex, basal 1/2 brownish yellow, apical 1/2 brownish black, with brownish black hairs; other flagellomeres cylindrical, brownish black with brownish black hairs; ultimate flagellomere 1.6 times length of penultimate flagellomere. Clypeus oval, brownish yellow, twice as long as the width; labrum brownish yellow; labellum brownish yellow with brown hairs; proboscis about 0.63 times length of head width. Palpus with five segments, 1^st^ segment almost invisible; 2^nd^ and 3^rd^ segments cylindrical, yellow with brown hairs; 4^th^ segment cylindrical, slightly swollen apically, basal 1/2 yellow, apical 1/2 brownish black, with brown hairs; 5^th^ segment slender, brownish yellow with brown hairs; relative length of distal four segments as 1.0: 1.0: 1.1: 2.3.

***Thorax*** (Fig. 4b) pruinose. Pronotum and propleuron brown without hairs. Mesonotum dark brown with middle area of posterior margin yellow; scutellum pale brown with anterior margin yellow, with numerous hairs grouped at posterolateral corner; metanotum brown; episternum brown; epimeron yellow. Relative length of femur, tibiae and 1^st^ to 5^th^ tarsomeres in fore leg as 15: 13: 7.4: 3.4: 2: 1: 1, in mid leg as 15.4: 12.8: 7.4: 2.6: 2.4: 1: 1, in hind leg as 23: 20: 7.2: 2: 1.4: 1: 1. Fore coxa pale with basal margin brownish yellow, with brownish yellow hairs; mid and hind coxae pale with brownish black hairs; trochanters pale, anterior margin with black spot apically, with brownish black hairs; femora yellow basally and gradually darkened apically, with brownish black hairs; fore and mid tibiae brown with brownish black hairs; hind tibia brownish yellow with brownish black hairs; tarsomeres brown with brownish black hairs; claw brown. Tibial spurs 0–0–0. Wing (Fig. 4c) slightly brown apically, apical 1/3 of sc brown; veins brown. Sc rudimentary, not ending at base of Rs; Rs slightly curved basally, as long as r-m; R_4_ wavy, the length from end of R_1_ to end of R_4_ shorter than length from end of R_4_ to end of R_5_; r-m straight, included angle between r-m and Rs less than 90 degrees; the length from end of M_1_ to end of M_2_ as long as the length from end of M_2_ to end of CuA_1_. Base of halter pale, apex of halter brown with brownish black hairs.

***Abdomen*.** First tergum brown with middle area pale, 2^nd^ tergum brown, 3^rd^ to 5^th^ terga brown with basal 1/3 brown, 6^th^ to 8^th^ terga brown; 1^st^ sternum pale, 2^nd^ to 6^th^ sterna pale with brown stripes laterally, 7^th^ sternum pale; abdomen with brown hairs. Male genitalia (Fig. 5c–f) brown. Epandrium trapeziform, posterior margin concave, with several brown hairs. Cercus triangular, inner margin bulge, with several brown hairs; anal cone round with two long hairs apically. Gonostylus slightly swollen and notched apically, outer side with a wide triangular lobe folded ventrally, with hairs. Gonocoxal lobe bifurcated, outer gonocoxal lobe transparent, rod-shaped, slightly curved; inner gonocoxal lobe transparent, rod-shaped, nearly straight, slenderer than outer gonocoxal lobe. Hypandrium rectangular, twice as long as the width, slightly narrow basally, posterior margin concave, with several brown hairs. Dorsal paramere with posterior margin round; dorsal carina apparent, tip nearly perpendicular.

**Female**. Body length 6.00 mm, wing length 7.50 mm, wing width 2.75 mm.

**Figure 6.**
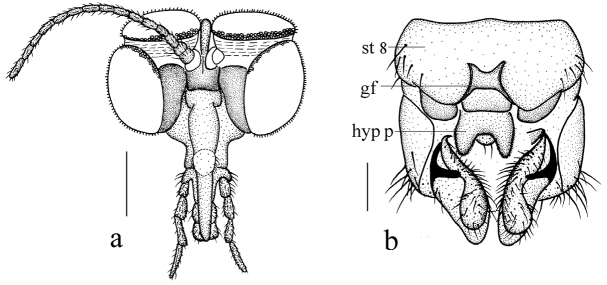
*Blepharicerabalangshana* sp. nov. **a** female head, frontal view **b** female terminal, ventral view. Scale bars: 0.25 mm (**a**); 0.10 mm (**b**). Abbreviations: gf = genital fork; hyp p = hypogynial plate; st 8 = eight sternite.

***Head*** (Fig. 6a) pruinose. Compound eyes subholoptic, interocular ridge present; each compound eye divided, callis oculi present; dorsal division separated from ventral division, as large as ventral division; dorsal division with about 20 rows of ommatidia, ommatidia red-orange, larger in diameter, with omatrichia; ventral division black with omatrichia. Scape oval, brown with brown hairs; pedicel conical, dark brown with brown hairs; first flagellomere constricted at base, flared at apex, basal 1/2 brownish yellow, apical 1/2 brownish black, with brownish black hairs; other flagellomeres cylindrical, tapering apically, brownish black with brownish black hairs; ultimate flagellomere 1.47 times length of penultimate flagellomere. Labrum brown; labellum pale with brown hairs; mandibles absent; proboscis about 0.74 times length of head width. Palpus with five segments, 1^st^ segment almost invisible, yellow with brownish black hairs; 2^nd^ segment cylindrical, yellow with brownish black hairs; 3^rd^ and 4^th^ segments cylindrical, brownish yellow with brownish black hairs; 5^th^ segment slender, cylindrical, brownish yellow with brownish black hairs; relative length of distal four segments as 1.0: 1.5: 1.5: 2.2. Tibial spurs 0–0–0. Terminalia (Fig. 6b): 8^th^ sternite bilobate, medial depression W-shaped, with six hairs laterally; genital fork X-shaped; hypogynial plate broad basally, bilobate posteriorly, each lobe round apically, intervalvular area U-shaped, with short hairs posteriorly; epiproct with two prominent hairs apically; spermathecae three in number.

##### Type material.

***Holotype***: male (CAU), China: Sichuan Province, Xiaojin County, Mount Balangshan, 2013.VII.9, 3281 m, Xiaoyan Liu; ***Paratypes***: 5 males 1 female (QAU), same data as holotype.

##### Distribution.

Currently known only from China (Sichuan).

##### Etymology.

The specific name refers to the type locality Mount Balangshan.

##### Remarks.

This new species is very similar to *B.indica* (Brunetti, 1911) from Afghanistan, Pakistan, Sri Lanka and India but can be separated by the apex of the gonostylus being slightly swollen and notched, the dorsal carina being apparent with nearly perpendicular tip. In *B.indica*, the apex of gonostylus is not swollen or notched, and the dorsal carina is inapparent (Zwick 1990). This new species is also similar to *B.asiatica* from Russia, Afghanistan, Pakistan, Sri Lanka and India, but it can be separated from the latter by the scutellum being pale brown with anterior margin yellow, the sterna of abdomen being mostly pale, and the dorsal carina with nearly perpendicular tip. In *B.asiatica*, the scutellum and the sterna of abdomen are dark brown, the dorsal carina has a very pointed and downcurved tip which is almost parallel to plate sometimes (Zwick 1990).

#### ﻿ Blepharicera kongsica sp. nov.

Taxon classificationAnimaliaDipteraBlephariceridae

E8D3DD9B-B540-5DE0-98A9-DFA3B0CDFF6E

http://zoobank.org/BFAAE688-7006-4834-9F59-67716BDEAAB7

Figs 7 , 8 , 9

##### Diagnosis.

Compound eye with dorsal division 1/15 as large as ventral division in male. Tibial spurs 0–0–2 in female. Rs 1.2 times as long as r-m. Cercus triangular. Dorsal branch of gonostylus short and broad, slightly swollen apically; ventral branch longer and slenderer than dorsal branch. Outer gonocoxal lobe transparent, S-shaped; inner gonocoxal lobe fusiform. Dorsal carina inapparent. Genital fork V-shaped.

##### Description.

**Male.** Body length 4.00–4.50 mm.

**Figure 7.**
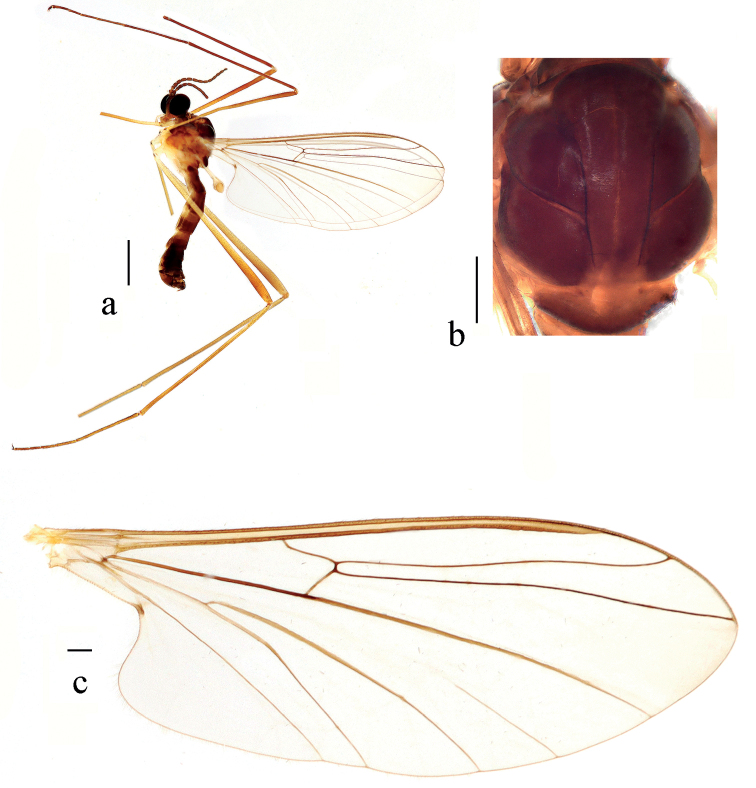
*Blepharicerakongsica* sp. nov. **a** habitus of male, lateral view **b** thorax, dorsal view **c** wing. Scale bars: 1.0 mm (**a**); 0.25 mm (**b, c**).

**Figure 8.**
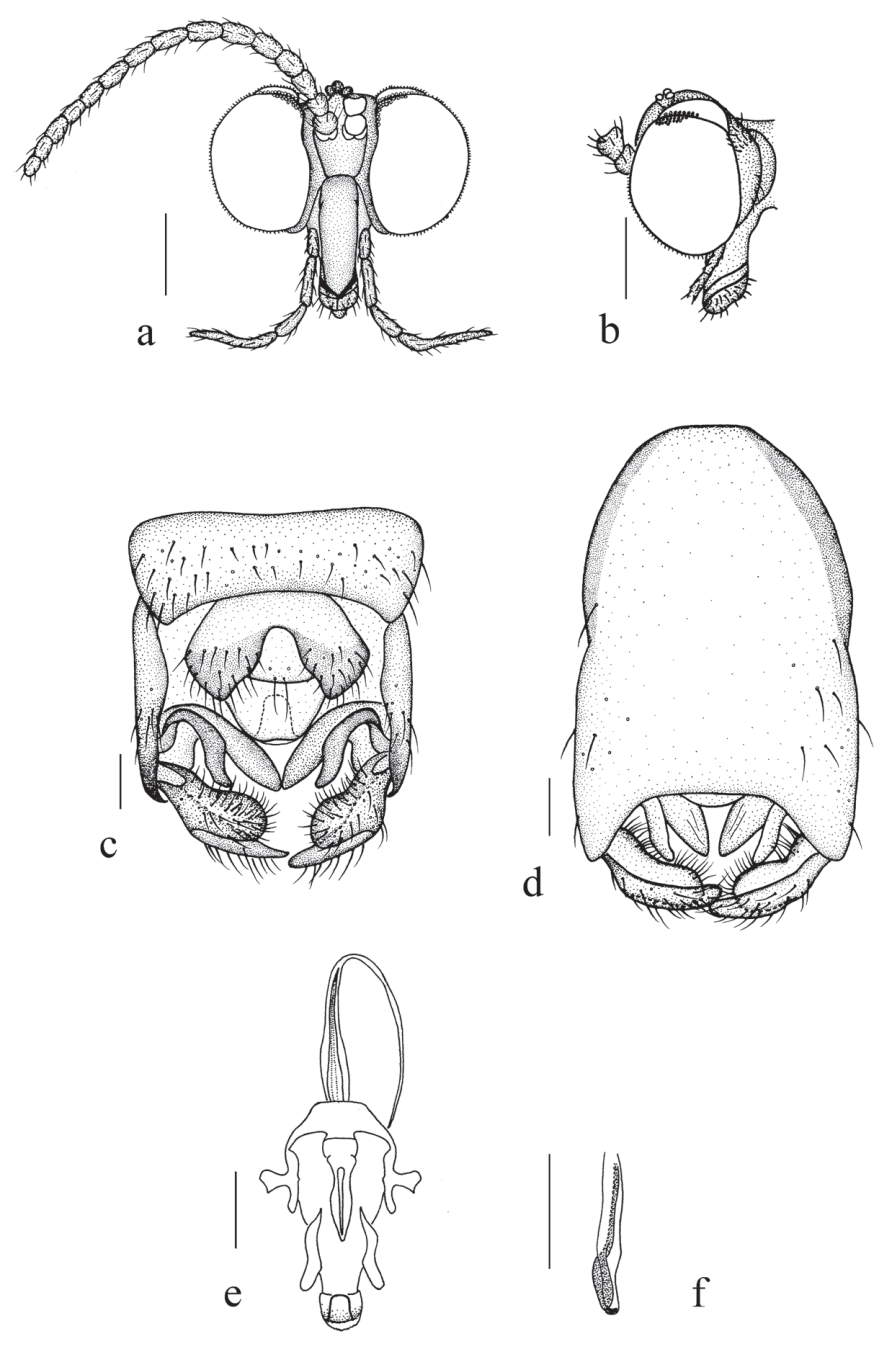
*Blepharicerakongsica* sp. nov. **a** male head, frontal view **b** male head, lateral view **c** male genitalia, dorsal view **d** male genitalia, ventral view **e** aedegal complex, dorsal view **f** tip of dorsal paramere, lateral view. Scale bars: 0.25 mm (**a, b**); 0.10 mm (**c–f**).

***Head*** (Figs 7a, 8a, b) pruinose, uniformly dark brown with dark brown hairs. Compound eyes dichoptic, interocular ridge absent; each compound eye divided, callis oculi absent; dorsal division contiguous with ventral division, 1/15 as large as ventral division; dorsal division with 7–8 rows of ommatidia, ommatidia red-orange, larger in diameter, with brown omatrichia; ventral division black with omatrichia. Ocelli brownish yellow. Scape and pedicel oval, brown with brownish black hairs; first flagellomere conical, basal 1/2 brownish yellow, apical 1/2 brownish black, with brownish black hairs; other flagellomeres cylindrical, dark brown with dark brown hairs; ultimate flagellomere 1.2 times length of penultimate flagellomere. Clypeus rectangular, basal 1/2 brown, apical 1/2 brownish yellow, twice as long as the width. labrum brownish yellow; labellum brownish yellow with dark brown hairs; proboscis about 0.56 times length of head width. Palpus with five segments, 1^st^ segment almost invisible; 2^nd^ to 4^th^ segments cylindrical, yellow with brown hairs; 5^th^ segment slender, yellow with dark brown hairs; relative length of distal four segments as 1.0: 1.7: 1.4: 3.2.

***Thorax*** (Fig. 7b) pruinose. Pronotum and propleuron dark brown without hairs. Mesonotum mostly dark brown, except middle area of posterior margin of scutum and middle area of scutellum light brown, scutellum with numerous hairs grouped at posterolateral corner; episternum dark brown; anepimeron yellow, katepimeron light brown. Relative length of femur, tibiae and 1^st^ to 5^th^ tarsomeres in mid leg as 10.0: 9.3: 5.3: 2.1: 1.3: 1: 1.3, in hind leg as 18: 15.8: 6.4: 2: 1.3: 1: 1.3. Fore coxa dark brown with dark brown hairs; mid and hind coxae pale with brownish black hairs; trochanters pale, anterior margin with black spot apically, with brownish black hairs; fore and mid femora brownish yellow basally and gradually darkened to dark brown apically, with dark brown hairs; hind femur yellow basally and gradually darkened to dark brown apically, with dark brown hairs; fore and mid tibiae dark brown with dark brown hairs; hind tibia brown with dark brown hairs. Tibial spurs 0–0–0. Wing (Fig. 7c) slightly brown apically; veins brown. Sc rudimentary, not ending at base of Rs; Rs slightly curved basally, 1.2 times as long as r-m; R_4_ wavy, the length from end of R_1_ to end of R_4_ shorter than length from end of R_4_ to end of R_5_; r-m straight, including angle between r-m and Rs less than 90 degrees; the length from end of M_1_ to end of M_2_ longer than the length from end of M_2_ to end of CuA_1_. Base of halter pale, apex of halter brown with dark brown hairs. Base of halter pale, apex of halter brownish yellow with dark brown hairs.

***Abdomen*.** First tergum brown with middle area pale, 2^nd^ tergum brown, 3^rd^ to 5^th^ terga brown with basal 1/2 light brown, 6^th^ to 8^th^ terga dark brown; 1^st^ sternum pale, 2^nd^ to 7^th^ sterna brown with brownish black stripes laterally; abdomen with dark brown hairs. Male genitalia (Fig. 8c–f) brown. Epandrium trapeziform, posterior margin concaved medially, with several brown hairs. Cercus triangular, inner margin bulge, with several brown hairs; anal cone flat with two long hairs apically. Gonostylus bifurcated, dorsal branch short and broad, slightly swollen apically, with hairs; ventral branch longer and slenderer than dorsal branch, with long hairs. Gonocoxal lobe bifurcated, outer gonocoxal lobe transparent, S-shaped, pointed apically; inner gonocoxal lobe fusiform, transparent. Hypandrium nearly rectangular, 1.5 times as long as the width, slightly narrow basally, posterior margin concave, with several brown hairs laterally. Dorsal paramere with posterior margin round; dorsal carina inapparent.

**Female**. Body length 5.50–6.00 mm, wing length 6.50–7.00 mm, wing width 2.25–2.50 mm.

**Figure 9.**
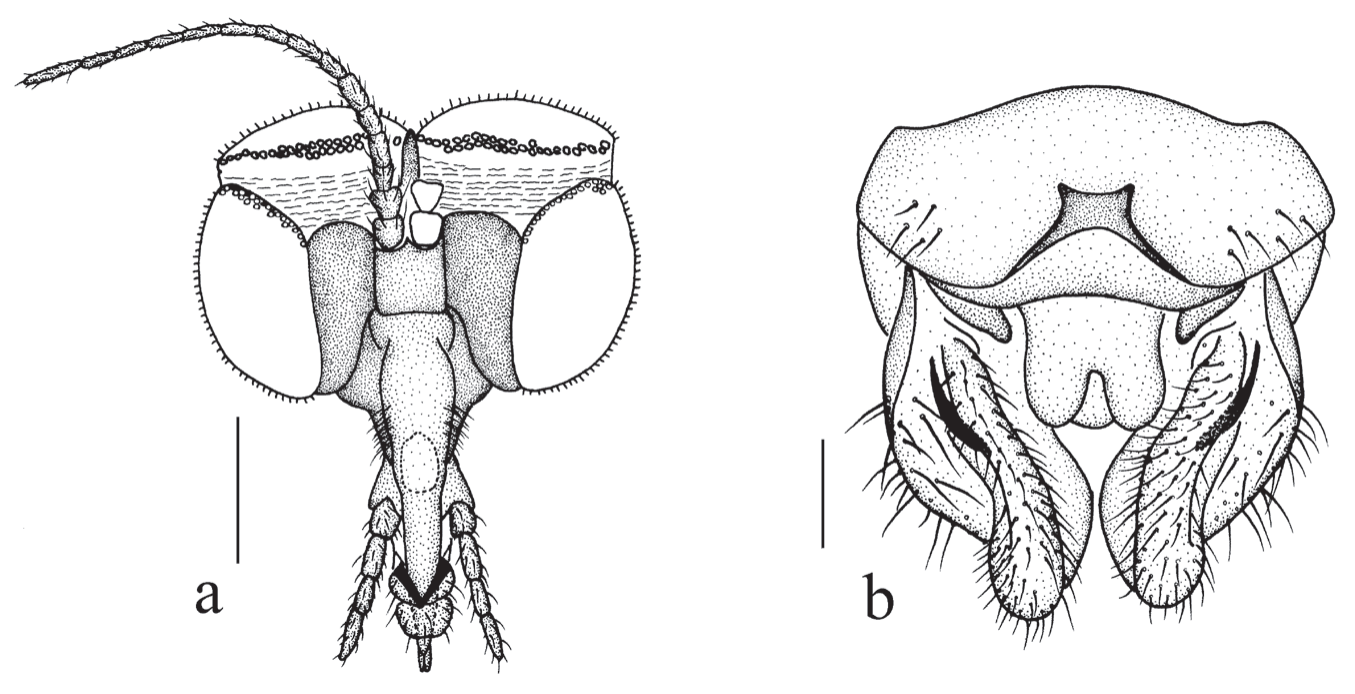
*Blepharicerakongsica* sp. nov. **a** female head, frontal view **b** female terminal, ventral view. Scale bars: 0.25 mm (**a**); 0.10 mm (**b**).

***Head*** (Fig. 9a) pruinose. Compound eyes subholoptic, interocular ridge present; each compound eye divided, callis oculi present; dorsal division separated from ventral division, as large as ventral division; dorsal division with about 14 rows of ommatidia, ommatidia red-orange, larger in diameter, with omatrichia; ventral division black with omatrichia. Scape oval, brownish black with brownish black hairs; pedicel conical, brownish black with brownish black hairs; first flagellomere constricted at base, flared at apex, basal 1/2 brown, apical 1/2 brownish black, with brownish black hairs; other flagellomeres cylindrical, tapering apically, brownish black with brownish black hairs; ultimate flagellomere 1.8 times length of penultimate flagellomere. Clypeus brownish black; labrum brown; labellum brownish yellow with brownish black hairs; mandibles brown; proboscis about 0.8 times length of head width. Palpus with five segments, 1^st^ segment almost invisible, brownish yellow with brownish black hairs; 2^nd^ to 5^th^ segments cylindrical, brownish yellow with brownish black hairs; relative length of distal four segments as 1.0: 1.2: 1.2: 1.5. Fore coxa dark brown with brownish black hairs; mid and hind coxae pale with brownish black hairs; trochanters pale, anterior margin with black spot apically, with brownish black hairs; fore and mid femora brownish yellow basally and gradually darkened to dark brown apically, with dark brown hairs; hind femur yellow basally and gradually darkened to dark brown apically, with dark brown hairs; fore and mid tibiae dark brown with brownish black hairs; hind tibia brown with brownish black hairs. Tibial spurs 0–0–2. Terminalia (Fig. 9b): 8^th^ sternite bilobate, medial depression broadly U-shaped, with several hairs laterally; genital fork V-shaped; hypogynial plate broad basally, bilobate posteriorly, each lobe round apically, intervalvular area U-shaped; spermathecae three in number.

##### Type material.

***Holotype***: male (CAU), China: Sichuan Province, Daofu County, Kongse, 2013.VIII.5, 2976 m, Xiaoyan Liu; ***Paratypes***: 1 male 7 females (QAU), same data as holotype.

##### Distribution.

Currently known only from China (Sichuan).

##### Etymology.

The specific name refers to the type locality Kongse.

##### Remarks.

This new species is very similar to *B.japonica* (Kitakami, 1931) from Japan but can be separated by the compound eyes being dichoptic in male and subholoptic in female, the facet of the dorsal division of the compound eye being larger than that of the ventral division, and the dorsal branch of the gonostylus being shorter than the ventral branch. In *B.japonica*, the compound eyes are broadly separated in both sexes, the facet of the dorsal division of the compound eye is smaller than that of the ventral division, and the dorsal branch of the gonostylus is longer than the ventral branch (Kitakami 1931; Zwick 1990). This new species is also similar to *B.fasciata* (Westwood, 1842) from Europe and Asia, but it can be separated from the latter by the dorsal division of the compound eye being contiguous with ventral division in male, the dorsal branch of the gonostylus being shorter than the ventral branch, and the concaved posterior margin of the hypandrium being flat. In *B.fasciata*, the compound eye has a narrow area between the dorsal and ventral divisions in male, the dorsal branch of the gonostylus is as long as the ventral branch, and the concaved posterior margin of the hypandrium is convex medially (Mannheims 1935; Zwick 1990).

## Supplementary Material

XML Treatment for Blepharicera gengdica

XML Treatment for Blepharicera balangshana

XML Treatment for Blepharicera kongsica
